# Detection of soluble urokinase type plasminogen activator receptors in children with gingivitis and normal subjects

**DOI:** 10.1186/s12903-022-02478-7

**Published:** 2022-10-03

**Authors:** Mohamed Abd‑Ellatif El‑Patal, Mona A. Khalil, Walaa Shipl, Ibrahim Barakat, Eman M. I. Youssef, Shahinaz El Attar, Adel Fathi, Alaa A. Abdallah

**Affiliations:** 1 Department of Pedodontics and Oral Health, College of Dentistry, Al-Azhar University, Cairo, Egypt; 2 Department of Biochemistry, Faculty of Medicine, Al-Azhar University, Cairo, Egypt; 3 Department of Biochemistry, College of Medicine, Taif University, Taif, Saudi Arabia; 4 Department of Pediatric Dentistry, College of Dentistry, Umm Al-Qura University, Makkah, Saudi Arabia

**Keywords:** Gingivitis, Salivary suPAR, Gingival index

## Abstract

**Background:**

Gingivitis is a reversible condition; however, if left untreated, it progresses to periodontitis, which a serious infection that leads to bone destruction. Soluble urokinase-type plasminogen activator receptor (suPAR) measurement may be of value in the early assessment of gingivitis in children, thereby minimizing risk of tooth loss.

**Objectives:**

In this observational study, we assessed salivary and serum concentrations of suPAR for the diagnosis of gingivitis and correlation of salivary suPAR with the periodontal clinical parameters.

**Methods:**

Ninety children participated in the study, with 20 healthy subjects as controls and 70 patients with gingivitis. The gingivitis group was divided into mild, moderate, and severe cases. According to the gingival index (GI), salivary and serum samples were analyzed for the suPAR and C-reactive protein levels using an enzyme-linked immunosorbent assay.

**Results:**

The salivary suPAR was significantly higher in patients with gingivitis (10.8 ± 2.9 ng/mL) than in the control group (7.0 ± 1.1 ng/mL) as *P* < 0.001. SuPAR was correlated with gingivitis severity. It was 7.7 ± 1.5 1 ng/mL in mild cases, 10.9 ± 1.2 ng/mL in moderate cases, and 14.4 ± 0.9 ng/mL in severe cases. The difference was significantly high (*P* < 0.001) between the groups; however, the difference between the mild cases and the control was nonsignificant as *P* < 0.066. The salivary suPAR was correlated with periodontal clinical parameters, which included GI and simple oral hygiene index (SOHI). Conversely the serum suPAR was not correlated with the salivary suPAR or the periodontal clinical parameters.

**Conclusion:**

The results of the present study demonstrated that the salivary suPAR is increased in proportionate with the degree of severity of gingivitis in children. Moreover, salivary suPAR was correlated with the periodontal clinical parameters.

## Introduction

Periodontal disease is one of the most common dental problems in both children and adults [1]. In children, it often manifests as gingivitis, which usually occurs as a result of bacterial plaque accumulation on the teeth cervical margins [2]. The body's reaction to oral microorganisms in dental plaque has been characterized by the production of different inflammatory and immune substances. These inflammatory substances play a major role in the periodontal disease progression [3]. Multiple studies have found that the estimation of these inflammatory biomarkers such as matrix metalloproteinases and cyto kines in different biological samples as saliva, serum, gingival tissues in patients with periodontal disease provide better understandings of the disease's pathogenesis [4, 5]. Also it could aid patient care by predicting diagnosing as in the study of Isola et al. [6] who found that periodontitis can be a significant predictor of both serum and salivary NLRP3 concentration,

The Soluble urokinase-type plasminogen activator receptor (suPAR) is a biological marker of inflammation and immune system activation [7]. SuPAR is a bioactive form of the urokinase plasminogen activator receptor (uPAR), which is a membrane-linked protein found in many immunologically active cells, including monocytes, neutrophils, activated T lymphocytes, macrophages and endothelial cells, keratinocytes, fibroblasts, smooth muscle cells, megakaryocytes, and certain tumor cells [8]. Polymorphonuclear neutrophils are one of the immune cells that affect the periodontal biofilm structure by destructing pathogens through production of oxidative substances and proteases combined with phagocytosis [9]. On of PMNs activation, uPAR is shed by several proteases leaving it devoid of glycosylphosphotidylinositol anchors to generate a soluble form. SuPAR has a stable three domain structure (D1, D2, and D3) that retains most of the uPAR activities, which are involved in cellular attachment, motility, and migration through its interaction with integrins [10].

suPAR is present in blood and other body fluids, such as cerebrospinal fluid, saliva, and urine [11]. High levels of plasma suPAR have been found in many diseases, such as diabetes, cancer, and rheumatoid arthritis [12, 13].

Skottrup et al. [14] evaluated the salivary suPAR levels in adolescents and found a positive association between salivary suPAR levels and clinical signs of periodontitis. Furthermore**, **Taşdemir et al. [15] also assessed the salivary suPAR levels in adults and found an increased suPAR level in periodontal disease, which may have a role in periodontal tissues inflammation.

To the best of our knowledge, this study represents the first estimation of salivary suPAR levels in children. As saliva is a noninvasive tool for assessing children’s health, we aimed to assess the inflammatory process that occurr locally in the oral cavity through measurement of salivary and serum suPAR levels in children with gingivitis and study its relationship with the clinical periodontal conditions and other inflammatory biomarkers, such as CRP.

## Subjects and methods

### Study design

The study involved90 children, consisting of 70 with gingivitis patients with different degrees of the disease and 20 children who were controls with healthy gingiva. The study protocol was approved by the local Committee of Ethics of the Faculty of Dentistry, Al-Azhar University Cairo, Egypt (No.618/2126), Additionally, the sharing depended on written informed consent and parental endorsement. The authors confirmed that all the methods were performed in accordance with the relevant guidelines and regulations.

The study was conducted between June and September 2019 in the Pedodontics Department of the Faculty of Dentistry, Al-Azhar University, Cairo, Egypt. The inclusion criteria included patients with healthy gingiva and those with gingivitis who are not more than 12 years old and have no periodontal therapy for the last 6 months.

The exclusion criteria included having any systemic disease, taking any treatment (anti-inflammatory, antibiotics, or anti-allergy drugs), and receiving any periodontal management for the last 6 months.

The clinical examination and assessment of the patients’periodontal conditions were performed. Löe and Silness’s gingival index [16] was used to record gingivitis severity. The scale ranged from 0.1 to 3.0 (mild gingivitis, 0.1–1.0; moderate gingivitis 1.1–2.0; and severe gingivitis, 2.1–3.0). Furthermore, the oral hygiene was estimated by examining of the dental plaque found on the inner and outer aspects of the six index teeth, which is in accordance with the criteria of Silness and Löe’s plaque index [17].

### Sample size analysis

#### Sample size calculation was performed using following formula

$$n \ge \left[ {\frac{{Z_{1 - \alpha } \times \sigma }}{E}} \right]^{2}$$

Based on previous study the level of suPAR in healthy control was 1.93 ± 2.13 while in gingivitis patients was 4.46 ± 3.76 Taşdemir et al. [15] using SD (σ) with margin of error (E) = 0.8 and 90% confidence level (α = 0.10) the required sample for healthy control was 20 participant and for gingivitis group was 62 patients and 10% of attrition was added to reach final sample size 70 gingivitis patients.

### suPAR and CRP measurment

Saliva and serum samples were collected from all subjects. The parents and children were instructed to avoid taking a big meal within 60 min of sample collection. The preceding oral hygiene procedure was performed the previous night, as it may have caused bleeding gums, contaminating the saliva with the blood. The participants were also instructed to wash their mouths with water to remove any residual food before the sample collection. Saliva was collected using paper strips and placed under the tongue on the floor of the mouth for 1–2 min. After collection, the paper strips were centrifuged at 1500 × *g* for 15 min. Three milliliters of blood were collected, and they were centrifuged again for 10 min after clotting. Saliva and serum samples were stored at 70 °C until the time of assay. Salivary and serum suPAR concentrations were analyzed using the Biotech Human suPAR ELISA kit (Kono Biotech, China), lot number 201701, catalog number KN2319 Hu). The salivary CRP was estimated using a Salimetrics CRP ELISA kit (USA), which has a very high sensitivity level and low detection limit of 10 pg/mL. The serum CRP concentration was estimated using the Oxis International Inc. (CA, USA), according to the manufacturer’s instructions.

### Statistical analysis

Statistical analyses were performed using the IBM SPSS Statistics version 20 (SPSS Inc., Chicago, IL, USA). The categorical data are presented as frequencies and percentages, while and the chi-square test was used the for comparisons between the groups. Continuous data are reported as the mean ± standard deviation. Student’s T-test was used for comparisons between gingivitis and control groups, Where continuous data were normally distributed. For the comparison between the types of gingivitis, the ANOVA test was performed and post-hoc analysis was conducted by using the least significant difference test. In all statistical tests, *p *value value < 0.05 was considered statistically significant in all statistical tests.

### Study outcome

Primary outcome was detection of suPAR level in children with gingivitis and in health controls.

Secondary outcome was detection of the severity gingivitis and correlation of salivary suPAR with periodontal clinical parameters.

## Results

### Clinical characters of the studied groups

**Fig. 1:**
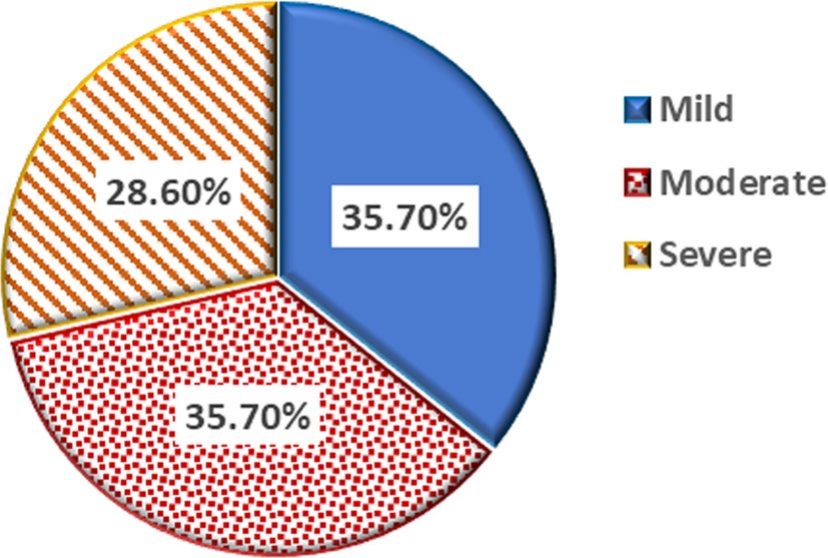
Gingival Index of cases

**Fig. 2:**
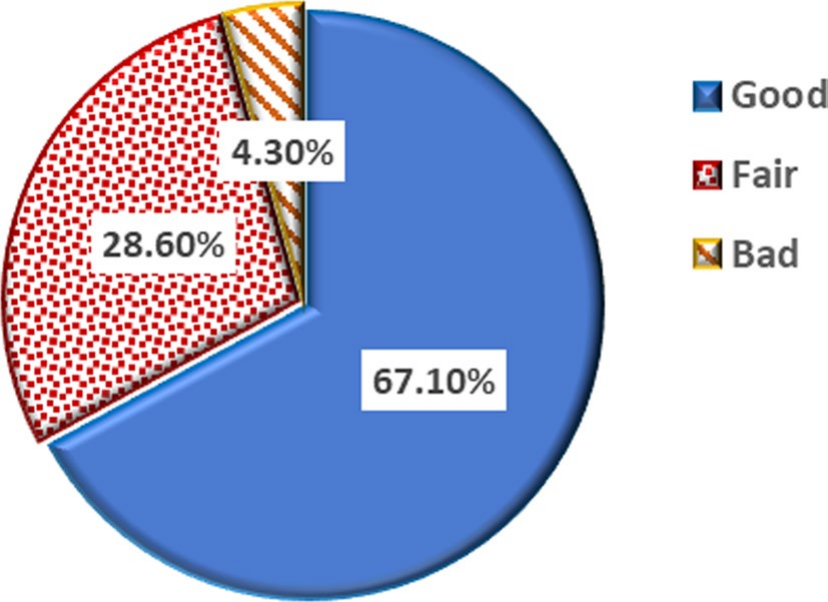
Simple oral hygiene index of cases

The study groups included children with a mean age of 8.7 ± 1.7 years, which ranged from 6 to 12 years in the gingivitis group and a mean age of 8.3 ± 1.6 years, which ranged from 6 to 11 years, in the control group as shown in Table 1. The GI was 0 in the control group. Conversely, the GI in the gingivitis group was 35.70% for mild cases (GI = 0.1–1.0), 35.70% for moderate cases (GI = 1.1–2.0), and 28.6% for severe cases (GI = 2.1–3.0), as shown in Fig. 1. The simple oral hygiene index (SOHI) was good in the control group, whereas in the gingivitis group it was good in 67.10% for mild cases, fair in 28.605% for moderate cases, and bad in 4.30% for severe cases in the gingivitis group, as shown in Fig. 2.

**Table 1:** Differences in parameters of studied groups

Variable		Cases (n = 70)	Control (n = 20)	*P* value*
Age (years)				
Mean ± SD		8.7 ± 1.7	8.3 ± 1.6	0.310
Sex				
Male		35 (50%)	10 (50%)	1.0
Female		35 (50%)	10 (50%)	
Salivary suPAR (ng/ml)				
Mean ± SD		10.8 ± 2.9	7.0 ± 1.1	< 0.001 **^**
Serum suPAR (ng/ml)				
Mean ± SD		2.3 ± 0.7	2.0 ± 0.7	0.064
Salivary CRP (pg/ml)				
Mean ± SD		728.0 ± 211.0	637.2 ± 141.1	0.074
Serum CRP (mg/l)				
Mean ± SD		3.2 ± 1.4	2.6 ± 1.7	0.095

Data presented as mean ± SD or number and percentage n(%)

*Student's T-test and Chi-square test were used

^Significant *p* value

### Descriptive statistical analysis

**Fig. 3:**
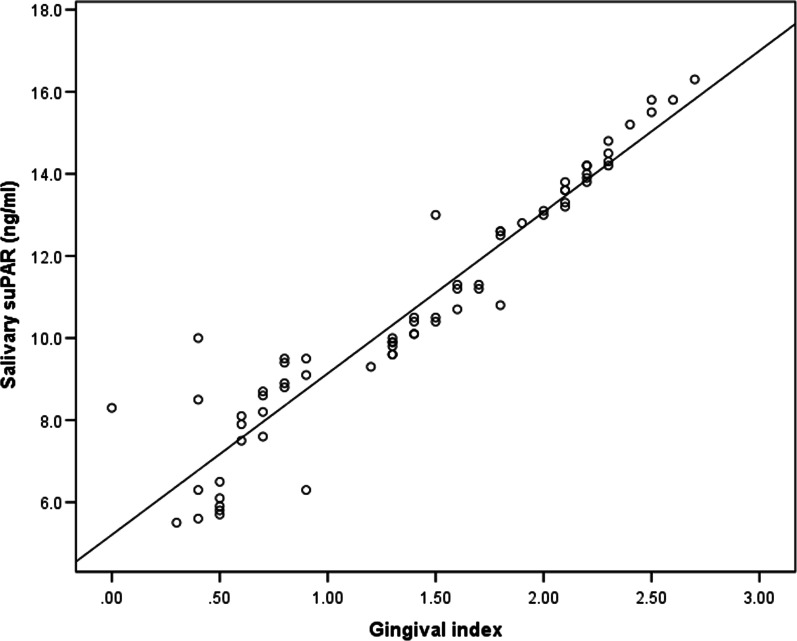
Correlation between salivary suPAR and GI in cases

The salivary suPAR level was significantly higher in the group of patients with gingivitis (10.8 ± 2.9 ng/mL) with a range of 5.5–16.3 than the control group (7.0 ± 1.1 ng/mL) with a range of 5.1–9 (*p* value was ˂ 0.001) as shown in Table 1. There was a highly significant difference in the salivary suPAR levels between the control group and the cases with moderate and severe gingivitis (*p* value was ˂ 0.001). However, with mild gingivitis cases, the p value was ˂ 0.066, as shown in Table 2. Additionally, the salivary suPAR levels differed between the subgroups of patients with gingivitis. The salivary suPAR level was 7.7 ± 1.5 ng/ml, 10.9 ± 1.2 ng/ml, and 14.4 ± 0.9 ng/ml in the mild, moderate, and severe gingivitis groups, respectively. The difference was significant (*P *˂ 0.001), as shown in Table 3. A significant difference was also found in the salivary suPAR level and degree of oral hygiene, as shown in Table 4. The salivary suPAR and GI were positively correlated; (r = 0.950, *p* < 0.001). However, no correlation between age or sex and salivary suPAR levels was found, as shown in Table 5 and Fig. 3.

**Table 2:** Post hoc analysis to compare control with gingivitis groups as regards salivary suPAR

	Mild	Moderate	Sever
Control	0.066	< 0.001 **^**	< 0.001**^**

**Table 3:** Comparison between Gingivitis patients subgroups Regarding all parameters

Variable		Mild (n = 25)	Moderate (n = 25)	Severe (n = 20)	*P* value*	P_1_	P_2_	P_3_
Age (years)		8.8 ± 1.8	9.1 ± 1.7	8.1 ± 1.5	0.123	0.559	0.143	0.046^
Sex								
Male		11 (44.0%)	13 (52.0%)	11 (55.0%)	0.741			
Female		14 (56.0%)	12 (48.0%)	9 (45.0%)				
Salivary suPAR (ng/ml)		7.7 ± 1.5	10.9 ± 1.2	14.4 ± 0.94	< 0.001 **^**	< 0.001^	< 0.001^	< 0.001^
Serum suPAR (ng/ml)		2.3 ± 0.57	2.4 ± 0.67	2.2 ± 0.81	0.624	0.592	0.645	0.335
Salivary CRP (pg/ml)		561.4 ± 157.2	763.1 ± 187.0	892.6 ± 140.3	< 0.001	< 0.001^	< 0.001^	0.011
Serum CRP (mg/l)		2.6 ± 0.83	3.6 ± 1.3	3.6 ± 1.7	0.009	0.006	0.011^	0.944

Data presented as mean ± SD or number and percentage n(%)

P1 Mild versus Moderate, P2 Mild versus Severe, P3 Moderate versus Severe

*One-way ANOVA with LSD post-hoc test (P_1_-P_3_) and Chi-square test were used

^Significant *p* value

**Table 4:** Relationship of Salivary and serum suPAR with different clinical parameters of cases

Parameter		Mean ± SD	*P *value*
*Salivary suPAR (ng/ml)*			
Sex			0.191
Male		11.2 ± 2.8	
Female		10.3 ± 3.0	
Gingival index			< 0.001 ^
Mild *(n* = *25)*		7.7 ± 1.5	
Moderate *(n* = *25)*		10.9 ± 1.2	
Severe *(n* = *20)*		14.4 ± 0.9	
SOHI			< 0.001 ^
Good *(n* = *47)*		9.2 ± 2.2	
Fair *(n* = *20)*		13.6 ± 1.1	
Bad *(n* = *3)*		16.0 ± 0.3	
*Serum suPAR (ng/ml)*			
Sex			0.213
Male		2.2 ± 0.7	
Female		2.4 ± 0.6	
Gingival index			0.624
Mild		2.3 ± 0.6	
Moderate		2.4 ± 0.7	
Severe		2.3 ± 0.8	
SOHI			0.355
Good		2.4 ± 0.6	
Fair		2.1 ± 0.8	
Bad		2.3 ± 1.1	

Data presented as mean ± SD

*Student's T-test was used

**Table 5:** Correlation of salivary and serum suPAR with age and GI in cases

	Correlation coefficient (r)	*P* Value *
*Salivary suPAR (ng/ml)*		
Age	− 0.033	0.786
Gingival index	0.950	< 0.001 **^**
*Serum suPAR (ng/ml)*		
Age	0.094	0.441
Gingival index	− 0.037	0.763

*Pearson’s correlation was used

**Fig. 4:**
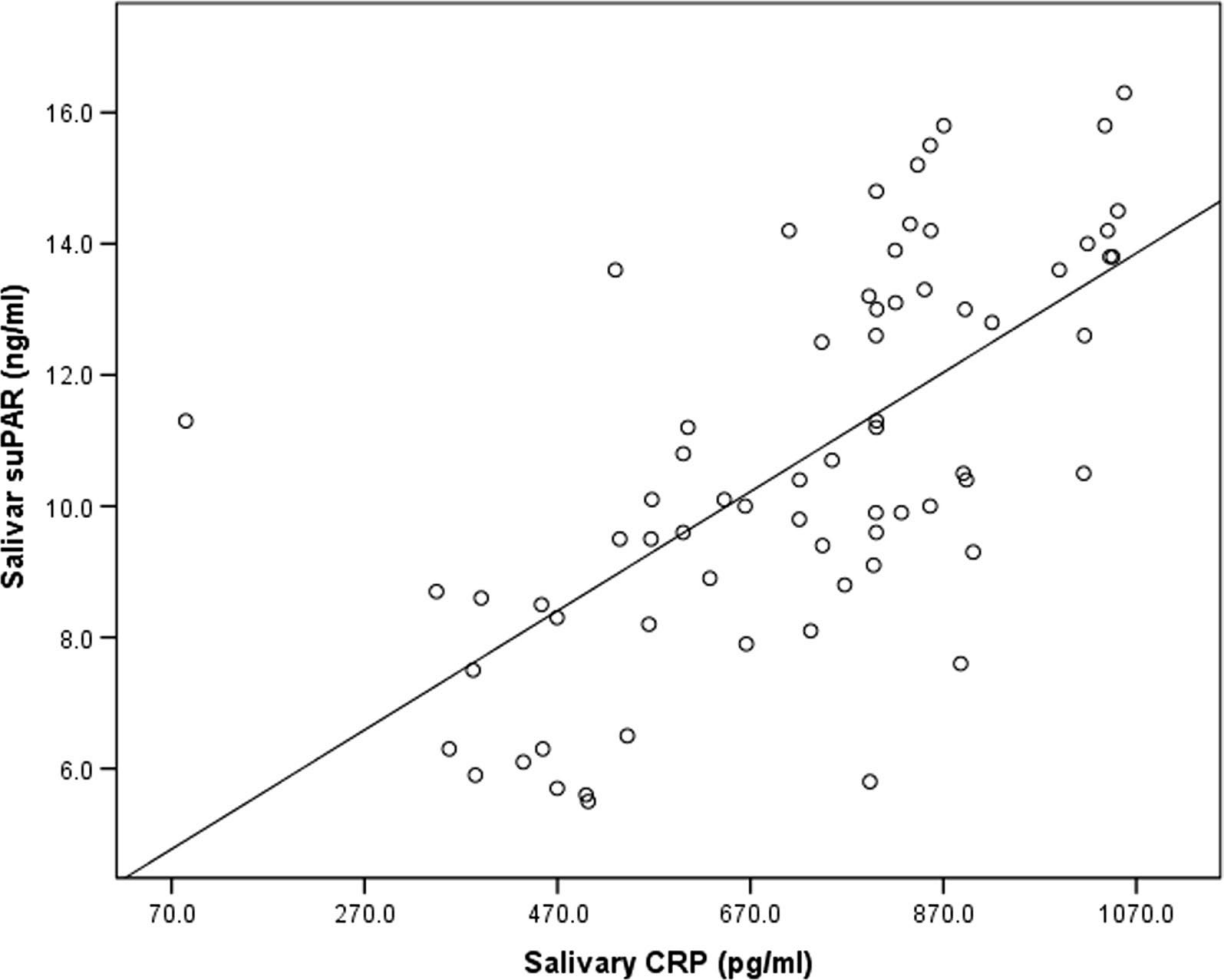
Correlation between salivary suPAR and salivary CRP in cases

**Fig. 5:**
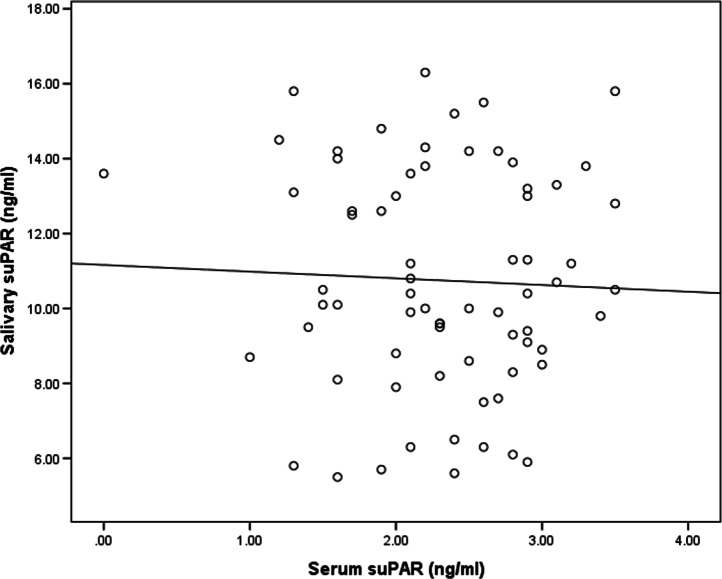
Correlation between salivary suPAR and serum suPAR in cases

**Fig. 6:**
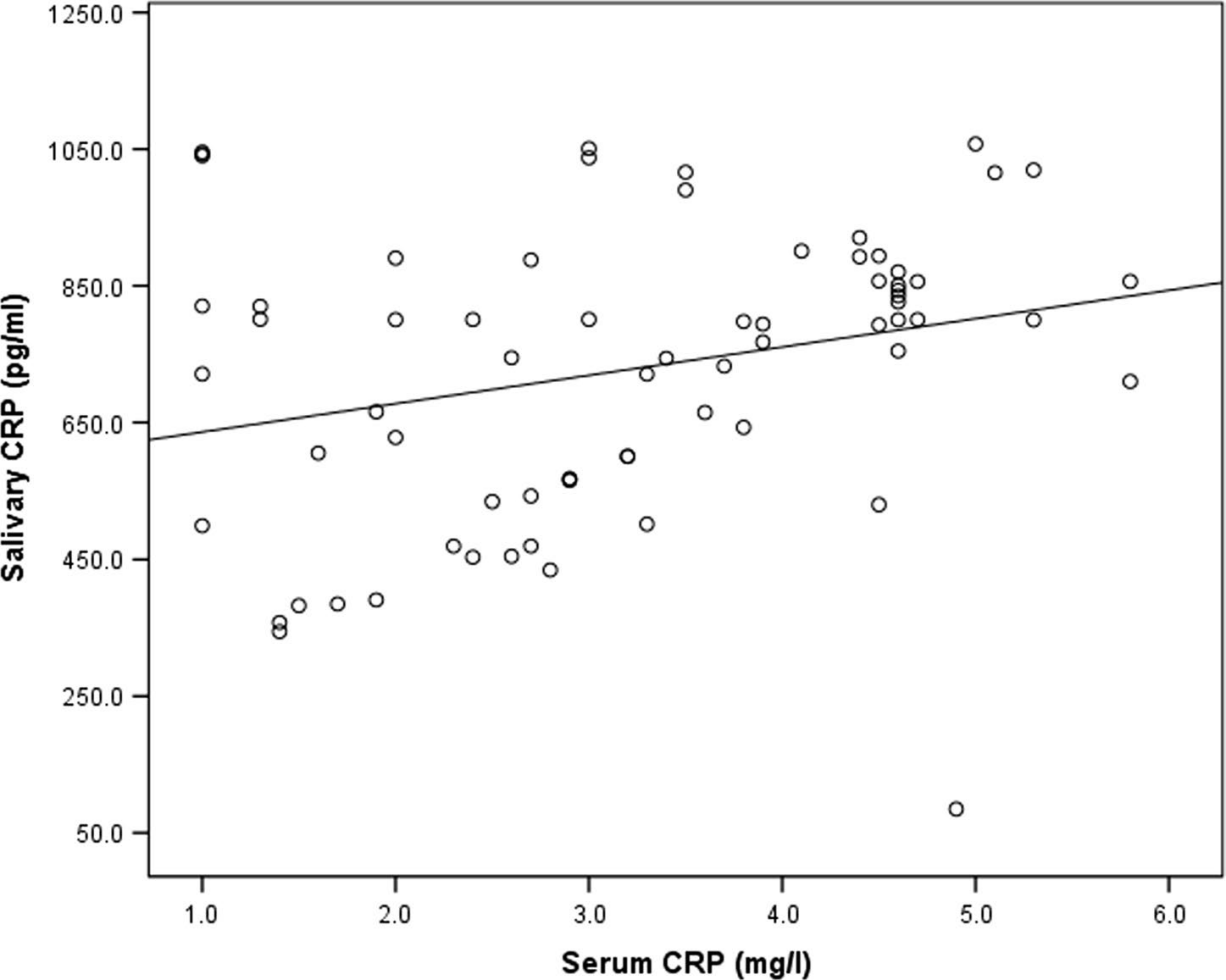
Correlation of serum CRP with serum suPAR and salivary CRP in studied groups

There was no significant difference in the serum suPAR level between the gingivitis and control groups. It was 2.3 ± 0.7 ng/ml in the gingivitis group and 2.0 ± 0.7 ng/mLin the control group, as shown in Table 1. With respect to periodontal conditions, no significant difference in serum suPAR levels was observed between patients with various degrees of gingivitis or oral hygiene, as shown in Tables 3 and 4. No correlation was found between the serum suPAR and salivary suPAR as shown in Table 6 and Fig. 4. Salivary CRP level was higher in the gingivitis group (793.4 pg/ml) than control (637.2 pg/ml) group, but the difference was not significant. Also, serum CRP was higher in the gingivitis group 3.2 ± 1.4 mg/l (1–5.8)] than in the control group [2.6 ± 1.7 mg/l (1.3–2.7)], but the difference was non-significant, as shown in Table 1. Additionally, there was significant difference in salivary CRP level between subgroups of gingivitis as shown in Table 3. A correlation was found between the salivary CRP and serum CRP levels as shown in Table 7 and Fig. 6. Moreover, a correlation was found between salivary suPAR and salivary CRP in the gingivitis group, as shown in Table 6 and Fig. 5. However, no correlation was found between serum suPAR and the serum CRP levels,as shown in Table 7 and Fig. 6.

**Table 6:** Correlation of Salivary suPAR with serum suPAR and Salivary CRP

	Salivary suPAR	
	Correlation coefficient (r)	*P* value *
*Serum suPAR*		
Cases	− 0.041	0.735
Control	0.316	0.174
*Salivary CRP*		
Cases	0.653	< 0.001 **^**
Control	0.047	0.844

*Pearson’s correlation was used

**Table 7:** Correlation of Serum CRP with serum Serum suPAR and Salivary CRP in studied groups

	Serum CRP	
	Correlation coefficient (r)	*P* value*
*Serum suPAR*		
Cases	0.137	0.196
Control	− 0.060	0.801
*Salivary CRP*		
Cases	0.265	0.026 **^**
Control	− 0.140	0.556

*Pearson’s correlation was used

## Discussion

Children with healthy gingiva may reach adulthood with good oral health [18]. Gingivitis improves with good treatment and continuous oral home care, whereas periodontitis is usually irreversible as it progresses, often leading to destruction of tooth supporting tissue and finally tooth loss [6]. Neglected gingivitis can progress to periodontitis. Therefore, early diagnosis of gingivitis, decreases the risk of tooth loss [19].

Children usually manifest periodontal diseases as gingivitis [20]. As saliva is a noninvasive, painless, and an important research tool for assessing the health of children, we aimed to assess salivary suPAR in children and study its relation to periodontal parameters and other inflammatory biomarkers such as CRP in our study.

In this study, the salivary suPAR levels in children with gingivitis were significantly higher than those in the control group. A significant difference in the salivary suPAR levels was found between the control group and patients with moderate and severe gingivitis, which indicated that salivary suPAR correlates with disease severity. This was in accordance with the study of Taşdemir et al. [15], who found that salivary suPAR was high in the periodontal disease group, which suggested that the salivary suPAR may be an important marker for the pathogenesis and development of gingivitis and periodontitis.

Furthermore, in our study, strong positive correlations were observed between the salivary suPAR and indices of periodontal conditions such as the GI and SOHI indices. These results concurred with those of the study conducted by Skottrup et al. [14], who found a positive association between the salivary suPAR level and the clinical signs of periodontitis, which suggested that inflammation in the oral cavity might be detected by estimating the salivary suPAR.

In the present study, salivary suPAR levels were found to be higher than serum suPAR levels and no relationship was found between the serum suPAR levels and indices of periodontal condition indices. This suggests that inflammation in gingival tissue was not strong enough to produce a systemic response that affected the serum suPAR levels. This was also supported by our finding that there was no correlation between the suPAR levels in the serum and saliva. The previous findings were in accordance with those of Gustafsson et al. [11], who found that the salivary suPAR levels were significantly higher than, but not correlated to, the plasma suPAR levels. Moreover, Skottrup et al. [14] found no correlation between the serum and salivary suPAR levels in a study of adolescents with periodontitis.

In this study, patients with gingivitis had higher salivary and serum CRP levels than those in the control group; however, the difference was not significant. The salivary CRP was in accordance with the study conducted by Shojaee et al. [21], which showed a considerable difference in CRP concentrations between the periodontitis group and healthy groups. Patients with gingivitis and healthy gingiva had lower CRP levels than those with chronic periodontitis.

Moreover, findings about the serum CRP concurred with those of Podzimek et al. [22], who found that the CRP levels were elevated subsequent to periodontal disease severity.

In this study, the suPAR and CRP levels were correlated in the saliva of the gingivitis group. This result contradicted that the study of Gustafsson et al. [11], who found no correlation in young adults with unknown periodontal conditions. Although multiple studies showed correlations between the CRP and suPAR in the blood that produced discordant results, no correlation was found between the CRP and suPAR in the blood. Hall et al. [23] found no correlation between theCRP and suPAR levels in the serum, Conversely, a study conducted by Slot et al. [24] showed that the suPAR level was correlated with CRP in patients with rheumatoid arthritis but not in patients with reactive arthritis. Additionally, a study done Isola et al. [25] found that periodontitis and high sensitivity CRP level were the only significant predictors of the augmented suPAR levels in the plasma and saliva, respectively.

In this study, a correlation was found between the serum and salivary CRP levels in the gingivitis group, which was consistent with a study by Ouellet-Morin et al. [26], who found an association between the CPR in saliva and serum, especially at elevated CRP levels (> 2.0 mg/L) that were found in the gingivitis group. Conversely, the study of Dillon et al. [27] found no association, which may be because the CPR level was measured in the group with healthy subjects. All the findings of this study clarified the clinical role of suPAR measurement in patients with gingivitis; however, a larger sample size, and prospective studies are needed to confirm our findings.

## Conclusion

The salivary suPAR was elevated proportionately with of gingivitis severity in children and was positively correlated with clinical parameters, including GI and SOHI Indices. The salivary suPAR may be considered a periodontal inflammatory biomarker and required further study to be more beneficial for the assessment of periodontal disease and therapy.

## Data Availability

All the data generated or analyzed during this study are included in this published article.

## Abbreviations

suPAR: Soluble urokinase-type plasminogen activator receptor
GI: Gingival index
SOHI: Simple oral hygiene index
CRP: C-reactive protein
